# Antioxidant and α‐glucosidase inhibitory capacity of nonextractable polyphenols in Mopan persimmon

**DOI:** 10.1002/fsn3.1314

**Published:** 2020-09-12

**Authors:** Chang Zhou, Kemin Mao, Jiao Li, Jie Gao, Xiaoyu Liu, Yaxin Sang

**Affiliations:** ^1^ College of Food Science and Technology Hebei Agricultural University Baoding China

**Keywords:** Antioxidant capacity, Anti‐α‐glucosidase, In vitro simulated digestion, Nonextractable polyphenol, Persimmon

## Abstract

This study was to evaluate and compare the polyphenols contents, antioxidant capacities, and α‐glucosidase inhibitory abilities of extractable and nonextractable polyphenols (EP and NEP) in Mopan persimmon. The results showed that total phenols content of NEP was 5 times higher than that of EP, and the hydrolyzed NEP compounds displayed higher antioxidant capacity than EP in vitro by DPPH, ORAC assays. Meanwhile, NEP also exhibited inhibition capacity of α‐glucosidase and were higher than that of acarbose. In addition, an in vitro model of gastrointestinal digestion was used for the release of NEP, the polyphenols content and ORAC values were obviously increased in gastric digestion stage. The result indicated that NEP in Mopan persimmon, which has often been overlooked and discarded in the past, possessed higher polyphenols content and antioxidant capacity than EP.

## Graphical abstract

Both EP and NEP in Mopan persimmon were evaluated for antioxidant and anti‐α‐glucosidase capacities. The NEP are the main contributor to the antioxidant capacity of Mopan persimmon. The NEP exhibited higher anti‐α‐glucosidase capacity than acarbose. Gastric digestion played a key role in the release of NEP.

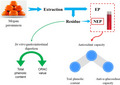

## 1 INTRODUCTION

Fruit has been considered as indispensable and beneficial food to human health because of their nutrient‐rich and convenient intake (Savran et al., 2016). The important natural antioxidants in fruit may reduce the risk of diseases such as cancer (Cohen, Kristal, & Stanford, 2016; Serafini et al., 2012), cardiovascular diseases (Aune et al., 2017; Mocan, Zengin, Crişan, & Mollica, 2016), inflammation (Koushki, Amiri‐Dashatan, Ahmadi, Abbaszadeh, & Rezaei‐Tavirani, 2018) and diabetes (Basu, Newman, Bryant, Lyons, & Betts, 2013; Bhooshan & Ibrahim, 2009). Though synthetic antioxidants such as butylated hydroxyanisole (BHA), butylated hydroxytoluene (BHT), tert‐butylhydroquinone (TBHQ) have been commonly added to food product to prevent or delay the oxidative deterioration. The demand for natural antioxidants has been gradually increased because of consumer's pursuit of health and environmental protection (Tang, Li, Chen, Guo, & Li, 2015; Yanishlieva, Marinova, & Pokorný, 2010).

Persimmons (*Diospyros Kaki* L. f) are widely cultivated in many countries around the world. Among them, the Mopan persimmon (*Diospyros Kaki* L. cv. Mopan), which is specially produced in China, has thousands of years of edible and medicinal history due to its high nutritional value (Chen, Fan, Yue, Wu, & Li, 2008). Apart from its nutritive value, persimmon fruits and leaves were long been used for medicinal purposes such as coughs, hypertension, dyspnoea, paralysis, burns and bleeding (Bo, Wu, Yu, Xiao, & Xu, 2017; Xie, Xie, Xu, & Yang, 2015). Recent studies indicated that persimmon possesses muti‐faced biological activities such as antioxidant (Haida & Hakiman, 2019) and anti‐adipogenic effects (Shin, Shon, Kim, & Lee, 2014), antitumor (Kim, Chung, Kim, & Kim, 2018; Park, Hwang, Hong, & Shin, 2017) and antidiabetic effects (Arakawa, Takasaki, Tajima, Fukamachi, & Igarashi, 2014). The polyphenols have been broadly verified to be the main compounds of persimmon that cause the above‐mentioned health effects (Su et al., 2017; Yongliang, Qingyu, Yan, & Liping, 2017). In particular, it was reported that persimmons contain considerable amounts of polyphenols compounds, such as chlorogenic acid, catechin, epicatechin, gallic acid, and proanthocyanidin (Ge, Zhu, Peng, Deng, & Li, 2016; Zhou et al., 2016), which contribute significantly to the total antioxidant activity of persimmon (Li, Lu, & Zhou, 2016).

Polyphenols content in fruits and its bioactive on health have been widespreadly investigated in past researches. But the main object of these researches was almost focused on the field of extractable polyphenols (EP) which can be extracted with aqueous‐organic solvents, whereas there was a significant amount of bioactive compound remained in the corresponding extraction residues which was called as nonextractable polyphenols (NEP) (Arranz, Silván, & Sauracalixto, 2010). Therefore, there would be an undervaluation of the phenolic contents of fruits as well as antioxidant activities if their NEP fraction of residue was not involved (Arranz, Sauracalixto, Shaha, & Kroon, 2009; Tow, Premier, Jing, & Ajlouni, 2011). So further study and more comprehensive data are needed to confirm persimmon may possess more polyphenols content and stronger biological activities such as antioxidant, prevention of diabetes.

The total production of persimmon in China was 396.9 tons in 2016, this ranked China first in the world. But what was inconsistent with it was that persimmons are perishable and difficult to store and transport, which resulted in a large number of persimmons being discarded, also brought inconvenience and restriction in processing. In China, Japan, Spain, and Italy, Mopan persimmons were often processed into fruit wine and vinegar (Zou et al., 2018), and the remaining residues which contain large amounts of NEP were wasted, resulting in the decline of yield and environmental problem. If these processing wastes can be fully utilized, the yield and health benefits of persimmon fruit products can be greatly improved.

In this study, the total polyphenols contents of EP and NEP in persimmon were evaluated, and the antioxidant and anti‐α‐glucosidase capacity of EP and NEP was also compared in vitro. In addition, an in vitro model of gastrointestinal tract was performed to the digestion of residue from persimmon, and the release of NEP was also investigated.

## 2 MATERIALS AND METHODS

### 2.1 Plant material

Mature and fully colored fruit of persimmons (*Diospyros Kaki* L. cv. Mopan) were harvested in December in Baoding, China and immediately stored at −20 ℃ for further processes.

### 2.2 Chemicals and reagents

Folin–Ciocalteu's phenol reagent, gallic acid, α‐glucosidase (100 U/mg), α‐amylase (13 U/mg), pepsin (3,000 U/mg), bile salts and trypsin (250 U/mg) were purchased from Shanghai yuanye Bio‐Technology Co., Ltd (Shanghai, China). 2,2‐Diphenyl‐1‐picrylhydrazyl (DPPH·), ascorbic acid, trolox (6‐Hydroxy‐2,5,7,8‐tetramethylchromane‐2‐carboxylic acid), 4‐nitrophenyl‐α‐D‐glucopyranoside (pNPG), dihydrochloride (AAPH), fluorescein sodium salt, and acarbose were purchased from Sigma‐Aldrich Chemical Co. All chemicals used were of analytical grade.

### 2.3 Sample preparation(Preparation of EP)

The extraction method used for EP from persimmon was according to the reported method (Zurita, Díazrubio, & Saura‐Calixto, 2012) with modification.

Fresh persimmons (1,027 g) were peeled and homogenized with 12 L of 90% aqueous ethanol, and the homogenization was led to ultrasonic‐assisted extraction for 30 min. After filtering, the filtrate was collected and another 12 L of 90% aqueous ethanol was added to the residue. After the extracting and filtering procedures were repeated two times, the combined filtrate extract was concentrated *in vacuum* decompression and lyophilized. Then, water and hexane were sequentially added to the extract, hexane‐soluble components were separated and collected, and then, ethyl acetate was added to the remaining aqueous solution for extraction. After the layers were separated, water‐soluble and ethyl acetate‐soluble components were obtained. All the obtained solution were concentrated *in vacuum* decompression and lyophilized to give EP, then stored at 4 ℃ for further analysis. The extracting residue was lyophilized to extract NEP.

### 2.4 Preparation of NEP

The extraction procedure of NEP from residue was according to the method described previously (Matsumura et al., 2016) with a slight modification.

NEP were extracted by acid hydrolysis using HCl. In brief, the lyophilized residue (1 g) was mixed properly with 10 ml of HCl and 10 ml of MeOH solution and heated at 90℃ for 4 hr. The hydrolysis solution was then adjusted to pH 5 using 1 mol/L NaOH and centrifuged at 6,000 *g* for 15 min at 4℃ to obtain the supernatant. Then, the supernantant was diluted to 100 ml with MeOH and stored at 4℃ for further analysis.

### 2.5 Determination of total phenol contents

The total phenol contents of each component (EP and NEP) were evaluated using the slightly modified Folin–Ciocalteu method (Singleton, Orthofer, & Lamuela‐Raventós, 1999). In brief, 1,000 μl of sample and 500 μl of Folin–Ciocalteu reagent were premixed with 4,500 μl of deionized water in a plug test tube and allowed to stand at room temperature for 10 min. Then, 4,000 μl of Na_2_CO_3_ (7.5% w/v) was added and the mixture was reacted for 2 hr at room temperature. The absorbance was measured at 765 nm. Different concentrations of gallic acid standard solutions were prepared for the generation of a standard curve. Results were expressed as milligrams of gallic acid equivalent (GAE) per gram of dry weight of persimmon extract (mg GAE/g dry weight (DW)).

### 2.6 DPPH radical scavenging activity assay

The scavenging activity against DPPH radical of EP and NEP was evaluated by the method (Amorim et al., 2019) with slight adjustment. In short, each concentration (40–200 μg/ml) of samples diluted with methanol were mixed and shaken appropriately with DPPH· (0.1 mM) dissolved in methanol. After reacting in dark at room temperature for 0.5 hr, the absorbance of mixture was measured at 517 nm using methanol as the blank, and ascorbic acid was used as the positive control. Radical scavenging activity was expressed as the scavenging rate and calculated using the following formula:

$$A\left(\%\right)=\left(1-\frac{A_{1}}{A_{0}}\right)\times 100\%$$

where A is DPPH· scavenging activity; *A_1_* and *A_0_* are the absorbance of samples and blank, respectively.

### 2.7 ORAC assay

The method for measuring ORAC values of each sample was carried out according to literature (Schaich, Tian, & Xie, 2015) with slight modification. The reaction was performed in 75 mM phosphate buffer (pH 7.4) with a final reaction volume of 200 μL and was assayed using a multi‐mode microplate reader. Briefly, samples (50 μL) and fluorescein sodium (50 μl, 126 nM) were placed in wells of the microplate and pre‐incubated at 37 ℃ for 10 min. AAPH solution (100 μl, 221 mM) was quickly added. The microplate was immediately placed in the reader, and fluorescence intensity values of each well were detected every 2 min for 60 min at an excitation wavelength of 485 nm and an emission wavelength of 535 nm. The same volume of phosphate buffer was used instead of the sample as a blank. The ORAC values of each sample were expressed as μmol of Trolox equivalent per g of each sample.

### 2.8 Determination of α‐glucosidase inhibition activity

The method of determination was based on the test condition of literature (Mcdougall et al., 2005) with slight adjustment. The reaction was performed in 0.1 M phosphate buffer (pH 6.8) with a final volume of 250 μl. In brief, 50 μl of sample solution and 50 μl of 20 /mL α‐glucosidase were added to the wells of microplate. While they were shaken properly and pre‐incubated for 15 min at 37℃, 50 μl of 10 mM pNPG solution was added and mixed thoroughly to start the reaction for 1 hr at 37℃, and then, the reaction was stopped by the addition of 100 μl 0.2 mM Na_2_CO_3_. Since pNPG can be hydrolyzed to produce glucose and pNP under the action of α‐glucosidase, the released pNP has a maximum absorption at 405 nm, and the absorbance was measured using a microplate reader. The α‐glucosidase inhibition rate of each sample was calculated according to the formula:

$$I\left(\%\right)=\left(1-\frac{A_{S}-A_{\text{SB}}}{A_{C}-A_{B}}\right)\times 100\%$$

where *I* is the α‐glucosidase inhibition; *A*_S_ is the absorbance of the sample; *A*_SB_ is the absorbance of the sample blank; *A*_C_ is the absorbance of the control; and *A*_B_ is the absorbance of the blank.

### 2.9 In vitro gastrointestinal digestion

The model of an in vitro gastrointestinal digestion was established according to previous method (Gayoso et al., 2016). The digestion process of residue after EP extraction involved three steps: oral digestion, gastric digestion, and intestinal digestion.

In the oral digestion stage, the lyophilized residues after EP extraction (500 mg) were mixed with 20 ml of 0.9% saline. Subsequently, 0.5 ml of α‐amylase solution (50 /mL in 1 mM CaCl_2_ solution) was added after adjusting pH to 5.0 with 1 M HCl NaHCO_3_, and the mixture was incubated at 37°C for 3 min with shaking properly. After incubation, the mixture was centrifuged to obtain supernatant.

In the gastric digestion, the resulting mixture after oral digestion was adjusted to pH 2.0 with 1 M HCl, and 2 ml pepsin solution (200 mg/ml in 0.1 M HCl) was added. Then, the mixture was incubated at 37°C for 2 hr in a incubator shaker. The resulting supernatant after centrifugation was also collected.

In the intestinal digestion, the resulting mixture after oral and gastric digestion was adjusted at pH 7.0 with 1 M NaHCO_3_ before addition of 5 ml pancreatin‐bile salts solution (200 mg/ml of pancreatin + 800 mg/ml of bile salts solution in 0.1 M NaHCO_3_). After incubation at 37°C for 2 hr, the supernatant was also centrifuged and collected.

After each digestion step, the supernatant was diluted to 25 ml with deionized water and stored at −20℃ for further analysis.

Release rate of NEP of persimmon residues in vitro digestion was calculated as following formula:

$$R\left(\%\right)=\frac{A_{D}}{A_{H}}\times 100\%$$

where *R* is the Release rate; *A*_D_ is the polyphenols contents or ORAC values of NEP after in vitro digestion; and *A*_H_ is the polyphenols contents or ORAC values of NEP after acid hydrolysis.

### 2.10 Statistical analysis

All experimental results were performed in triplicate and expressed as mean values and standard deviations (mean ± *SD*, *n* = 3).

## 3 RESULT AND DISCUSSION

### 3.1 Total polyphenols content of EP and NEP

Since the researches of fruit and vegetable polyphenols mostly corresponded to the phenolic components analyzed in aqueous and organic extracts (extractable polyphenols), a considerable amount of potential bioactive polyphenols (nonextractable polyphenols) that remained in solid residues was ignored. NEP are high molecular weight proanthocyanidins and phenolic acids, usually associate with proteins, polysaccharides, and dietary fiber in food matrix (Saura‐Calixto, 2012). They were generally not included in polyphenols analysis, although the polyphenol content of NEP was even higher than that of EP. Therefore, several organic solvents (It has been determined that the n‐hexane component did not contain phenolic contents but few carotenoids) were used to extract EP, and acidified methanol was used for NEP extraction.

The yields and total phenolic contents (TPC) of EP and NEP are shown in Table. 1. In terms of yield of freeze‐dried extract from Mopan persimmon, the total amount of EP (71.50 g/100 g) was about 2.5 times higher than that of NEP (28.50 g/100 g), but the polyphenols content of NEP (218.30 mg/g) was far more than that of EP (36.07 mg/g). And by calculating the sum of the polyphenols content in EP and NEP components of the whole persimmon fruit, we can clearly know the contribution of EP and NEP to TPC in persimmon fruit was 20.81% and 79.19%, respectively. These data indicated that a large proportion of polyphenols remained in the residue after EP extraction, even though these residues account for a relatively small proportion (28.5%) of the weight of persimmon extract. Similar, most studies have suggested that several common foodstuff such as apple, peach, onion, walnut contained more NEP than EP (Saura‐Calixto, 2012). It was also reported that hydrolysis of black tea residue with 2.9% (w/w) enzyme at 45°C and pH 5.4 for 98 min improved the liberation of NEP offering fivefold higher extract yield (g/100 g) as compared with nontreated residue (Mushtaq et al., 2017). In general, conventional extraction reagents such as ethanol, methanol, and water produced an incomplete extraction by the impossibility of recovering NEPs due to strong interactions of these compounds with the matrix (protein, fiber, sugar, etc.). Thus, after extraction, a residue treatment was necessary for releasing NEPs trapped in the matrix using acid, alkaline, or enzymatic hydrolysis. By reducing the pH of the solution, acid hydrolysis method could help to break down noncovalent complex bonds between the analyte and the matrix in order to release NEP, which increased the polyphenol concentration of the analyte solution (Cheng et al., 2014). Therefore, these polyphenols in residue should be recycled and utilized to investigate their physiological activities.

**Table 1:** Yields, total phenolic contents, DPPH free radical scavenging capacities and ORAC values of EP and NEP in persimmon

Sample	Yield (g DW)	Contents of polyphenol		DPPH· scavenging activity		ORAC value	
		Total phenols^a^	Total amount	Ascorbic acid equivalent^b^	Total amount	Trolox equivalent^c^	Total amount
Extact of persimmon	100		7,856.2^d^		56,164.8^e^		69794^f^
ethyl acetate component	10.87	11.10 ±0.84c	120.7	140.8 ± 7.2c	1,530.9	92 ± 5c	1,000
Water component	60.63	24.97 ± 3.1b	1513.9	509.1 ± 11.1b	30,864.9	132 ± 10b	8,003
NEP	28.50	218.30 ± 15.70a	6,221.6	834 ± 20.0a	23,769.0	2,133 ± 102a	60,791

Note

Values in the same column with different letters presented significant differences (*p* < .01).

a Total phenols of each sample were expressed as milligrams of gallic acid equivalent (GAE) per gram of dry weight; Data were expressed as means ± *SD* of three experiments.

b DPPH· scavenging activities of each sample were expressed as milligrams of ascorbic acid equivalent per gram of dry weight; Data were expressed as means ± *SD* of three experiments.

c ORAC values of each sample were expressed as μmol of Trolox equivalent per gram of dry weight; Data were expressed as means ± *SD* of three experiments.

d Sum of the contents of polyphenols for each component.

e Sum of the DPPH· scavenging activities for each component.

f Sum of the ORAC values for each component.

### 3.2 Antioxidant capacity

**Figure 1:**
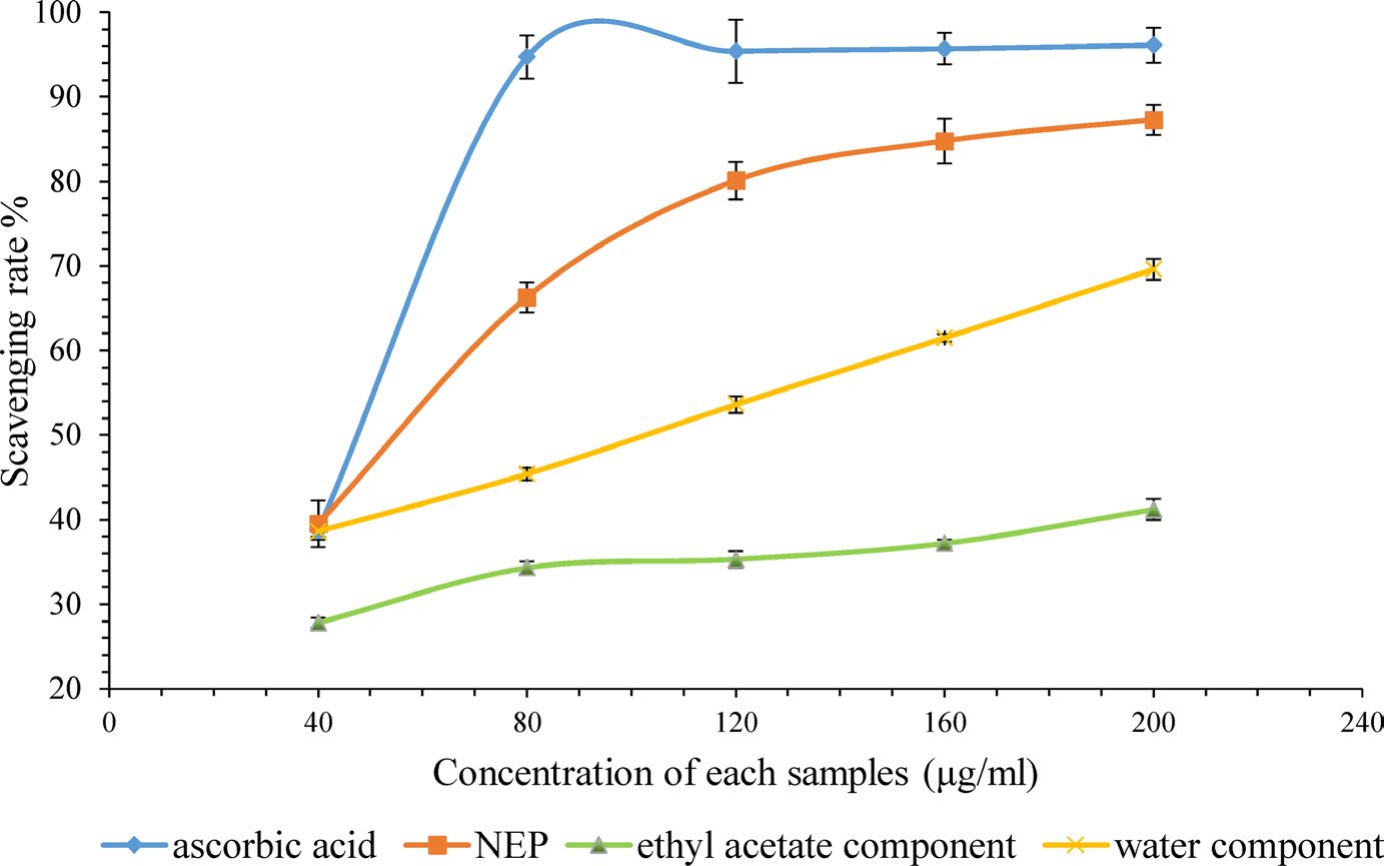
DPPH**·** scavenging activity of EP, NEP and ascorbic acid at each concentration

Due to the huge amount of NEP in persimmon, we can expect it may have a great antioxidant potential. The antioxidant capacities of EP and NEP were evaluated by methods including DPPH· and ORAC assay, and results were presented in Table 1. In both assays, NEP showed stronger antioxidant capacity than EP. Scavenging DPPH· activity of NEP with ascorbic acid equivalent of 834.0 mg/g was higher than that of EP with ascorbic acid equivalent of 649.9 mg/g. According to Figure 1, the scavenging rate of NEP (87.3 ± 0.7%) at concentration of 200 μg/ml was slightly lower than that of ascorbic acid (96.1 ± 1.6%) (*p* < .05), while that of EP (69.6 ± 0.5%) was significantly lower than them (*p* < .01). The ORAC value of NEP with Trolox equivalent of 2,133 μmol/g was about 9 times higher than that of EP with Trolox equivalent of 224 μmol/g. These data indicated that the phenolic compound in NEP had higher antioxidant capacity than that in EP, it was also observed in other literature (Peng, Li, Li, Deng, & Zhang, 2017).

In previous study, it was demonstrated that persimmon contained insoluble condensed tannins with high antioxidant potential (Huang et al., 2016). These condensed tannin may contain macromolecular proanthocyanidins, which can be dissociated with dietary fiber, polysaccharide, and protein by acid hydrolysis or enzyme digestion to release the polyphenols (Domínguezrodríguez, Marina, & Plaza, 2017) with antioxidant capacity. It was reported that the thiolysis degradation products of condensed tannins in persimmon consist of (epi) gallocatechin, epigallocatechin‐3‐O‐gallate and epicatechin‐3‐O‐gallate with a mean degree of polymerization ranging between 55 and 10 (Tian et al., 2012). In addition, the high molecular weight condensed tannin in persimmon residue mainly consisted of (epi) gallocatechin, epigallocatechin‐3‐O‐gallate, and epicatechin‐3‐O‐gallate, these were different from the polyphenols in EP which were predominantly consisted of catechins, gallic acid, and flavan‐3‐ol units (Tian et al., 2012). Higher molecular weight tannin was more effective antioxidants than were monomers or dimers. In addition, on an equimolar basis, the antioxidant activities of grape seed procyanidins are positively related to their degree of polymerization (Spranger, Sun, Mateus, Freitas, & Ricardo‐Da‐Silva, 2008).

According to Table 1, contribution of NEP to the sum of antioxidant capacity in persimmon extract was 42.3% for DPPH radical scavenging activity and 87.1% for ORAC value. It has been proved that the correlation between TPC and ORAC value is high (Escobedo‐Flores, Chavez‐Flores, Salmeron, Molina‐Guerrero, & Perez‐Vega, 2018); however, for the DPPH free radical scavenging assay, there are some limitations that lead to the inconsistency of TPC and DPPH· scavenging ability of NEP. NEP with complex structures and higher molecular weight more readily get in the way of each other and impede access to DPPH, they strongly block reaction at high concentrations (Schaich et al., 2015). Therefore, the DPPH· scavenging ability of NEP did not exhibit a good correlation with TPC. Overall, these data also reflected that NEP not only possessed more polyphenols content in persimmon, but also dominated antioxidant capacity in vitro, even if the yield of NEP was lower than that of EP. This result indicated that NEP was the significant antioxidant polyphenol compound in persimmon fruit.

### 3.3 α‐glucosidase inhibition activity

α‐glucosidase is a key enzyme that affects the digestion and absorption of carbohydrates in the diet. Inhibiting its activity can delay the absorption of glucose by the body, thereby inhibiting the rapid rise of postprandial blood glucose and preventing diabetes. Many studies have shown that polyphenols, flavonoids, and alkaloids could inhibit the activity of α‐glucosidase (Fwmad et al., 2017; Nasu, Miura, & Gomyo, 2005), but the inhibition of persimmon polyphenols on this enzyme has rarely been reported. Therefore, the inhibition effects on α‐glucosidase of EP and NEP in persimmon were evaluated in this study.

**Figure 2:**
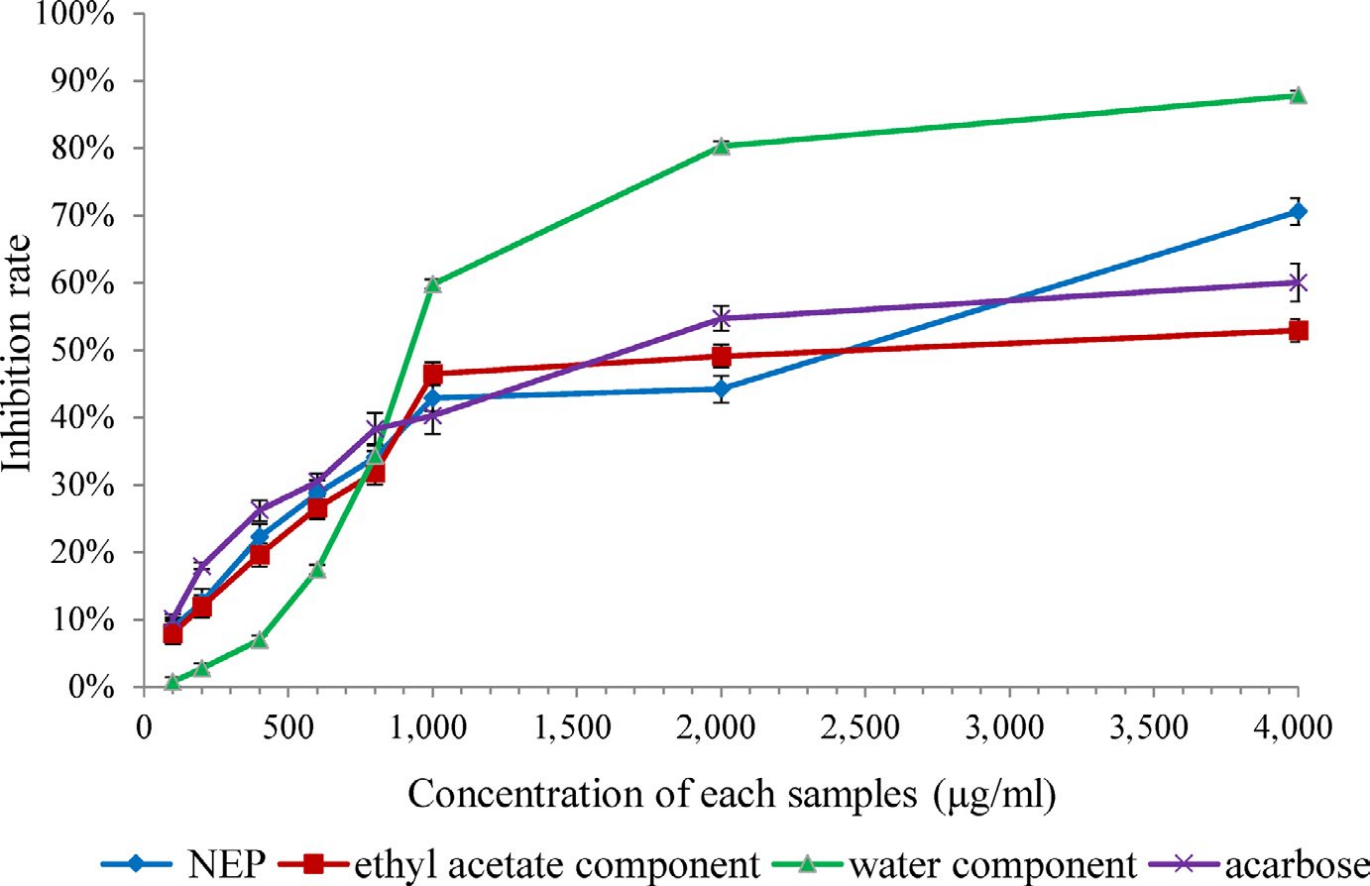
Inhibition activity of EP, NEP, and acarbose on α‐glucosidase

The inhibition effects of different concentrations of EP and NEP components on α‐glucosidase are shown in Figure 2. When the concentration was 4,000 μg/ml, the inhibition rate of the water extract component can reach 87.83%, while that of NEP was 70.62%. The inhibitory abilities of EP and NEP were compared by calculating the IC_50_ values of each component. The IC_50_ values of water component, ethyl acetate component, NEP, and acarbose were 1,082, 2,301, 1583, and 1806 μg/ml, respectively. These results showed that the inhibition ability of NEP was not as good as that of EP (*p* < .01), but it was still significantly stronger than that of acarbose (*p* < .05). However, this was inconsistent with the results of TPC and antioxidant capacities of EP and NEP. These differences were likely to result from the variations in phenolic composition and structure. Gallic acid, which can be extracted by ethanol, was the main phenolic component in EP (EtOH extract) of Mopan persimmon (Chen et al., 2008). It has been reported that gallic acid had a great inhibitory effect on α‐glucosidase (Oboh, Ogunsuyi, Ogunbadejo, & Adefegha, 2016). In addition, higher molecular weight anthocyanins such as cyanidin and delphinidin had lower inhibition of α‐glucosidase, but some lower molecular weight phenolic compounds such as flavan‐3‐ols, flavonones, and catechin which can easily bound into the active site of enzyme have stronger inhibition ability (Rasouli, Hosseini‐Ghazvini, Adibi, & Khodarahmi, 2017). Therefore, EP containing more of such low molecular weight phenolic compounds has higher inhibition ability against α‐glucosidase, while NEP exhibit lower inhibition ability. However, due to the possible side effects of synthetic hypoglycemic drugs, consumers are more inclined to safe and nontoxic natural drugs (Tundis, Loizzo, & Menichini, 2010), so NEP in persimmon may have great implication value of inhibiting α‐glucosidase.

### 3.4 The release of NEP and its antioxidant activity during in vitro gastrointestinal digestion

In the above, we had extracted NEP from the residue by acid hydrolysis using HCl‐MeOH solution and verified the content and antioxidant activity of NEP. However, the digestion and release of NEP from persimmon residue in the gastrointestinal fluid environment remained unclear, and studies on these have not yet appeared. Therefore, residue after EP extraction from persimmon was subjected to three in vitro simulated digestion procedures (oral, stomach, intestine digestion), and the polyphenols contents and ORAC values of digestion mixtures at each procedure were determined.

**Figure 3:**
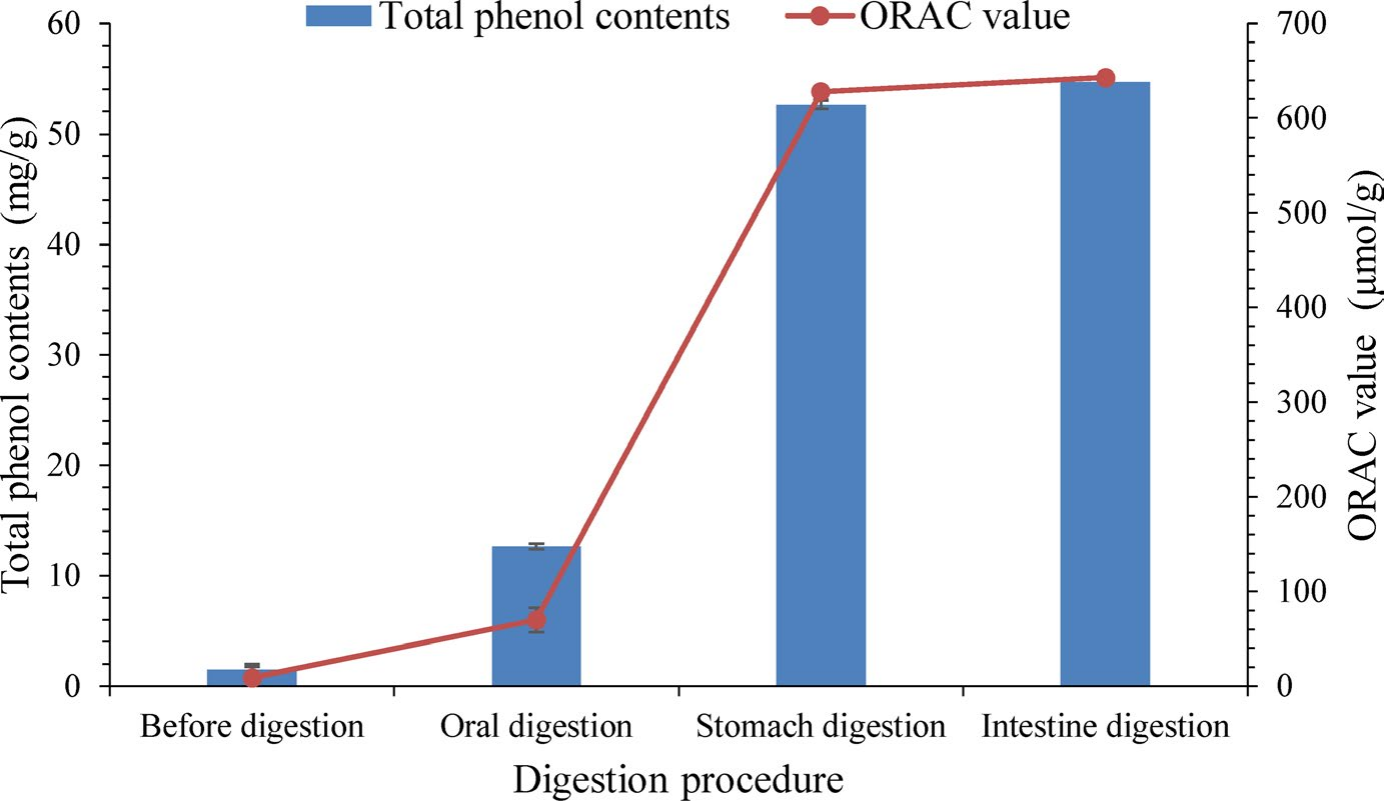
Polyphenols content and ORAC value of digestion mixture

As shown in Figure 3 and Table 2, different polyphenols content and ORAC values were observed at each stage of digestion. The polyphenols content and ORAC value of digestion mixture in oral procedure were lower, and increased significantly in stomach digestion (*p* < .01), but rose slightly in intestine digestion procedure (*p* > .05). These data suggested that pepsin and gastric acid played a leading role in the release of polyphenols during gastric digestion, which together promoted the release of polyphenols with antioxidant capacity. After pepsin and trypsin hydrolyzed protein, the polyphenols covalently or noncovalently bound to protein was released (Bouayed, Hoffmann, & Bohn, 2011). Most polyphenols bind to polysaccharides or proteins of food matrix in the form of hydrogen bonds, hydrophobic bonds, and ester bonds, while the acidic conditions of stomach environment can destroy these bonds and enhance the release of polyphenols (Liyana‐Pathirana & Shahidi, 2005). The possible reason that the polyphenols contents and ORAC values rose less during the intestinal digestion stage was that polyphenols such as phenolic acid, proanthocyanidins, which are unstable, degrade in the alkaline environment of intestinal fluid (Bouayed et al., 2011). After the whole digestion procedure, the release rate of NEP and its ORAC value in persimmon residue was 25.1% and 30.1%, respectively. It was indicated that NEP had a certain degree of release through in vitro simulated gastrointestinal digestion, and the released polyphenols with antioxidant activity can play an important role in gastrointestinal tract. However, the bioavailability of NEP, specifically the affect of intestinal microorganism on NEP release need to be further revealed.

**Table 2:** Release of NEP from digestion mixtures

	Polyphenols content (mg/g DW)	Release rate (%)	ORAC value (μmol/g)	Release rate (%)
Before digestion a	1.43 ± 0.08c	/	9 ± 1.4c	/
Oral digestion	12.65 ± 0.37b	5.8	70 ± 5b	3.3
Stomach digestion	52.65 ± 0.27a	24.1	628 ± 13a	29.4
Intestine digestion	54.70 ± 0.40a	25.1	643 ± 5a	30.1

Note

Values in the same column with different letters presented significant differences (*p* < .05).

a The lyophilized residue (500 mg) after EP extraction was mixed with 20 ml of 0.9% saline and directly subjected to TPC and ORAC determination.

## 4 CONCLUSION

In this study, nonextractable polyphenols (NEP) of persimmon exhibited higher polyphenol contents and antioxidant capacity in vitro including DPPH· scavenging activity and ORAC value than extractable polyphenols (EP), and the release of polyphenols and ORAC value of NEP during an in vitro gastrointestinal digestion were also evaluated. These results showed that NEP which was seldom focused constituted a major part of dietary polyphenols of persimmon fruit and possessed more important antioxidant capacity than EP. NEP also exhibited inhibition capacity of α‐glucosidase and was higher than that of acarbose. Furthermore, NEP were released from persimmon residues with a certain level after simulated gastrointestinal digestion, and the gastric digestion played a key role in the release of NEP.

In summary, NEP were the most effective antioxidant in persimmon fruit. This finding provided a favorable information for the full utilization and comprehensive analysis of polyphenols in Mopan persimmons. However, further researches were required to elucidate the composition and structure of phenolic compounds in EP and NEP to clarify their differences in biological activities.

## CONFLICT OF INTEREST

The authors have declared no conflict of interest.

## ETHICAL STATEMENT

This research did not include any human subjects and animal experiments.
